# Adenosquamous Carcinoma of the Stomach: A Population-based Study from the SEER Database

**DOI:** 10.7150/jca.29162

**Published:** 2019-09-07

**Authors:** Yugang Ge, Linling Lin, Xiang Ma, Dakui Luo, Liang Shi, Mingkun Jiang, Hao Fan, Yu He, Li Yang, Zekuan Xu

**Affiliations:** 1 Department of General Surgery, The First Affiliated Hospital of Nanjing Medical University, Nanjing, Jiangsu Province, China; 2 Department of General Surgery, The Second Affiliated Hospital of Nanjing Medical University, Nanjing, Jiangsu, China; 3 Department of General Surgery, Liyang People's Hospital, Liyang Branch Hospital of Jiangsu Province Hospital, Liyang, Jiangsu Province, China

**Keywords:** adenosquamous carcinoma, stomach, prognosis, SEER database

## Abstract

**Purpose**: Gastric adenosquamous carcinoma (ASC) is a rare pathological type with poorly understood clinicopathological features. The purpose of this study is to identify the characteristics of gastric ASC patients.

**Methods**: Using the Surveillance, Epidemiology, and End Results (SEER) database (2000 to 2014), patients with ASC (N=93) or adenocarcinoma (AC) (N=41794) of the stomach were included. The epidemiology, tumor features, treatment, and outcomes between these two groups were compared.

**Results**: The incidences of ASC from 1983 to 2014 [annual percentage change (APC) = -3.5%, 95% confidence interval (CI) -4.9 to -2.1] and AC from 1973-2014 [APC = -1.8%, 95%CI -2.0 to -1.6] decreased over time**.** Compared to AC cases, patients with ASC were more likely to present poor differentiation (74.2% vs 52.4%) and later summary stage (distant: 46.2% vs 33.6%) or later T stage (T4: 15.1%% vs 9.0%). Besides, the proportion of patients with distant metastasis (33.3% vs 23.9%), and chemotherapy (44.1% vs 34.0%) in ASC group was higher. The Kaplan-Meier analyses showed ASC cases had worse overall survival (OS) (*p*=0.017) than that of AC after propensity score matching (PSM), but not the cancer-specific survival (CSS) (*p*=0.849). The further subgroup analyses suggested no statistical significance between gastric ASC patients and AC patients for CSS. The multivariate cox proportional hazard analyses indicated that patients with distant summary stage (HR=2.11, *p*=0.014), no surgery (HR=2.22, *p*=0.016), and no/unknown chemotherapy (HR=3.33, *p*<0.001) were associated with poor OS for ASC population alone. However, for CSS, only ASC cases with no/unknown chemotherapy (HR=2.22, *p*=0.018) indicated worse outcomes.

**Conclusions**: Gastric ASC presented more aggressive clinicopathologic characteristics and poorer OS compared with AC. The localized/regional summary stages and undergoing surgery suggested favorable OS for gastric ASC patients. ASC cases receiving chemotherary showed both better OS and CSS.

## Introduction

In all pathologic types of gastric cancer, adenocarcinoma (AC) is the most common, whereas the incidence of adenosquamous carcinoma (ASC) is extremely low, accounting for less than 1% of total gastric malignancies 1, 2. ASC is a mixed neoplasia composed of AC and squamous cell carcinoma (SCC) components, with the latter making up at least 25% of the tumor mass 3. Some previous studies indicated that its biological behaviors were generally determined by the AC component, but the results remained controversial 4-6.

Compared with the traditional AC, gastric ASC usually had aggressive clinicopathologic characteristics, such as larger tumor size, deeper tumor depth, lymph node invasion, and poorer survival 7, 8. However, due to its rarity, most ASC information was based on case reports or small single-institution studies, so that these results were not very convincing. Therefore, this study was conducted to describe the epidemiology, tumor features, treatments, and outcomes between patients with ASC and AC via Surveillance, Epidemiology, and End Results (SEER) database. In addition, the risk factors influencing the prognosis of gastric ASC patients were analyzed.

## Material and Methods

### Study population

Supported by the National Cancer Institute, the SEER program gathers information of 18 population-based registered cancer institutes, which covers around 28% of the US population. According to the International Classification of Disease for Oncology, 3rd edition (ICD-0-3), we selected patients with gastric AC (SEER codes 8140-8145, 8210, 8211, 8214, 8220, 8221, 8255, 8260-8263, 8310, 8480, 8481, 8570, and 8574-8576) 9 and ASC (SEER code 8560) 10 during 2000 and 2014. All patients' data regarding the age, gender, race, marital status, primary site, grade, summary stage, T stage, lymph node metastasis, distant metastasis, surgery, radiation, and chemotherapy were extracted from the database. The endpoints were overall survival (OS) and cancer-specific survival (CSS). OS was defined as the time interval from diagnosis to death due to any cause, and CSS was the follow-up time from diagnosis to death due to gastric cancer. No personal identifying data were obtained from the SEER database. This study was approved by the review board at the First Affiliated Hospital of Nanjing Medical University.

### Statistical analysis

The SEER*Stat software, version 8.3.5 was used to estimate the age-adjusted incidence as diagnoses per 100, 000 patients per year. The Joinpoint software, version 4.6.0 was performed to calculate the annual percentage changes (APCs) for assessing the changes of incidence. The GraphPad Prism 6 software was adopted to draw the figure of incidence. The Chi-square test was conducted to compare the categorical variables. The Student's t-test was used to compare the continuous variables satisfying the normal distribution and homogeneity of variance, otherwise, the Mann-Whitney U test was performed. The Kaplan-Meier method and log-rank test were used to draw survival curves and evaluate the differences of OS and CSS between gastric ASC and AC patients, or for ASC patients alone by each covariate. The univariate and multivariable Cox proportional hazards models were carried out to determine the independent prognostic factors and the results were exhibited by the hazard ratio (HR) and 95% confidence interval (95% CI). We generated the 1:1 matched ASC group and AC group via a propensity score matching (PSM) method, reducing the effects of differences in baseline features. The characteristics for matching ASC and AC were age, race, grade, summary stage, T stage, distant metastasis, surgery, and chemotherapy. The statistical analyses were completed by SPSS 22.0, and R softwares. The *p* value <0.05 was considered to be significant.

## Results

### Population features

**Figure 1:**
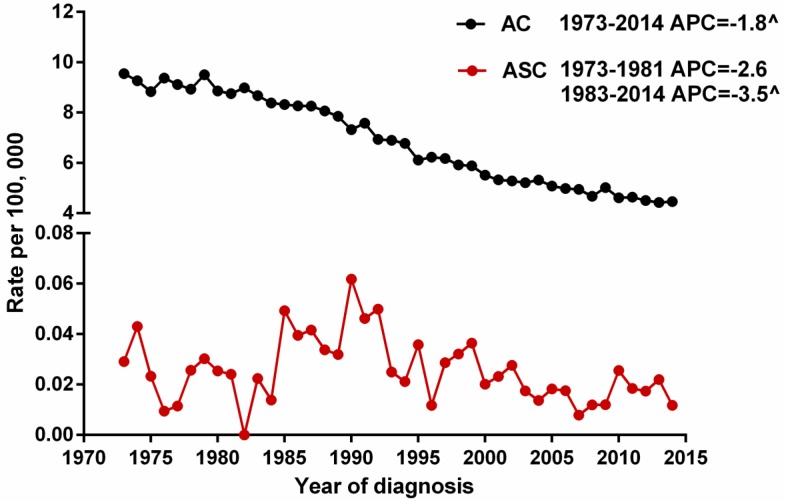
The age-adjusted incidences of gastric ASC and AC patients between 1973 and 2014 from the SEER database. ^indicated that the APC is significantly different from zero at the alpha = 0.05 level. ASC: adenosquamous carcinoma; AC: adenocarcinoma; APC: annual percent change.

We selected gastric ASC and AC patients from the SEER database during 1973 and 2014 to describe the incidence of these two histological types. Because the Joinpoint software cannot process records with dependent variable = 0 (the incidence of ASC at 1982 is 0), we calculated the APCs of ASC from 1973 to 1981 and from 1983 to 2014. The results showed that the age-adjusted incidences of ASC (1983-2014) and AC (1973-2014) significantly decreased over time (*p*<0.05), with the APCs of -3.5% [95% CI -4.9 to -2.1] and -1.8% [95% CI -2.0 to -1.6], respectively. However, there was no distinct decreased trend for the incidence of ASC during 1973 and 1981 (Figure 1).

Given that the treatment modalities, perioperative care, surgical methods and devices, and even the diagnostic ability change a lot, which will cause bias and affect the results. So, we extracted the data of ASC and AC patients between 2000 and 2014 from the SEER database. As a result, a total of 41887 gastric cancer patients including 93 ASC cases and 41794 AC cases were identified. The clinical characteristics of these subjects were summarized in Table 1. No remarkable difference was found between the two groups in terms of age, gender, ethnicity, marital status, primary site, lymph node metastasis, surgery, and radiation. ASC tumors tended to present a later summary stage (Distant: 46.2% vs 33.6%, *p*=0.002), a later T stage (T4: 15.1% vs 9.0%, *p*=0.036), and a higher distant metastasis rate (33.3% vs 23.9%, *p*=0.026) when compared to those with AC. Also, the ASC group was obviously correlative with a higher tumor grade (poor: 74.2% vs 52.4%; undifferentiated: 2.2% vs 1.5%, *p*=0.002) and more likely to receive chemotherapy (44.1% vs 34.0%, *p*=0.048).

### OS and CSS analyses

**Figure 2:**
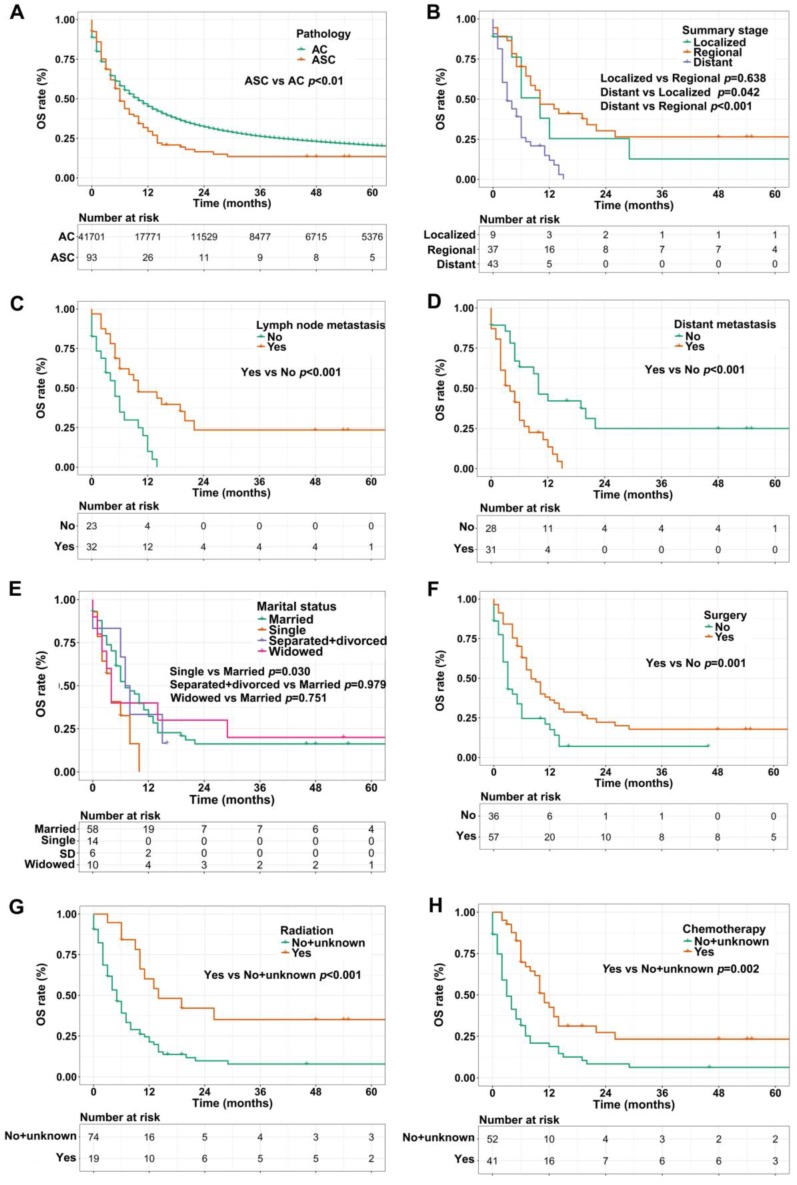
** (A)** OS for patients with gastric ASC and AC; **(B)** OS for ASC patients with distant, regional, and localized stages; **(C)** OS for ASC patients with lymph node metastasis or not; **(D)** OS for ASC patients with distant metastasis or not; **(E)** OS for ASC patients with different marital statuses; **(F)** OS for ASC patients receiving surgery or not; **(G)** OS for ASC patients receiving radiation or no/unknown; **(H)** OS for ASC patients receiving chemotherapy or no/unknown. ASC: adenosquamous carcinoma; AC: adenocarcinoma; OS: overall survival; SD: separated/divorced.

We used the Kaplan-Meier method to evaluate OS and CSS among gastric ASC and AC patients, excluding the cases with unknown survival time. As showed in Figure 2A, patients with ASC showed worse OS than AC patients (1-, 3-, and 5-year OS: 28.0% vs 42.6%, 9.7% vs 20.3%, 5.4% vs 12.9%, respectively, *p*<0.01). In the ASC group alone, compared to the cases with localized or regional disease, patients in distant summary stage didn't survive more than 2 years, having poorer OS (*p*=0.042 and *p*<0.001, respectively, Figure 2B). Interestingly, lymph node metastasis was significantly correlated to better OS (*p*<0.001, Figure 2C), however, ASC patients with distant metastasis remained worse OS (*p*<0.001, Figure 2D). Figure 2E exhibited that single ASC patients had a shorter OS than married (*p*=0.030). Furthermore, surgery, radiation, and chemotherapy could improve OS of ASC patients (*p*=0.001, *p*<0.001, and* p*=0.002, respectively, Figure 2F-H).

**Figure 3:**
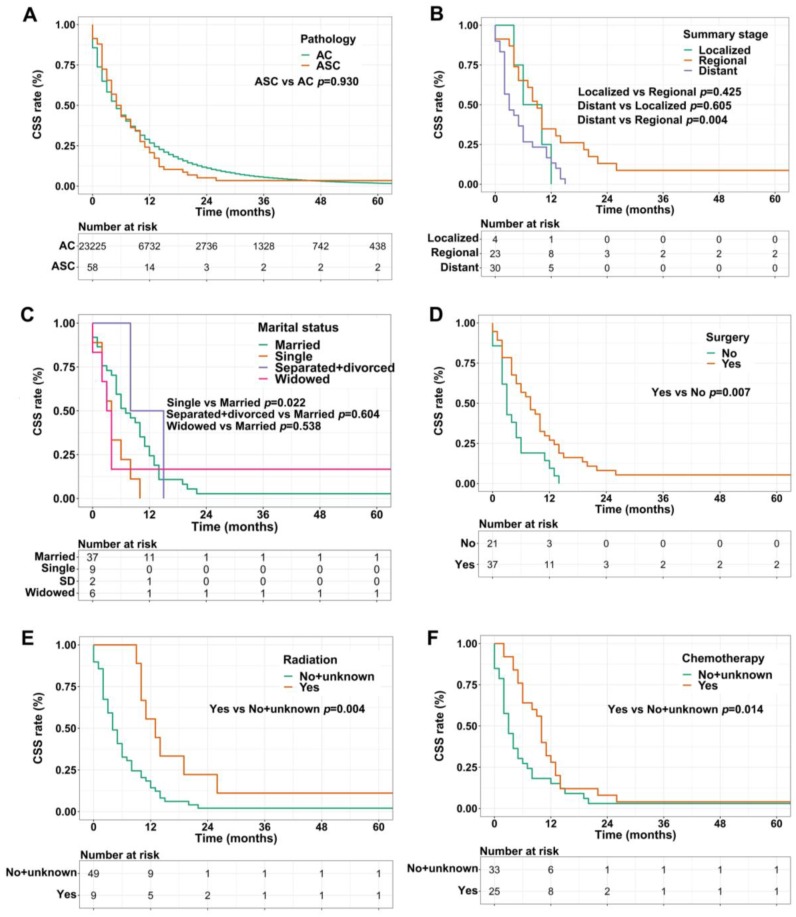
** (A)** CSS for patients with gastric ASC and AC; **(B)** CSS for ASC patients with distant, regional, and localized stages; **(C)** CSS for ASC patients with different marital statuses; **(D)** CSS for ASC patients receiving surgery or not; **(E)** CSS for ASC patients receiving radiation or no/unknown; **(F)** CSS for ASC patients receiving chemotherapy or no/unknown. ASC: adenosquamous carcinoma; AC: adenocarcinoma; CSS: cancer specific survival; SD: separated/divorced.

For CSS analysis, unexpectedly, there was no statistical difference between ASC patients and AC patients (*p*=0.930, Figure 3A). Similarly, in the ASC group alone, patients with distant summary stage presented worse CSS than those with regional disease (*p*=0.004, Figure 3B). The influences of marital status on CSS were consistent with the results of OS, that is, single ASC patients had shorter CSS than married (*p*=0.022, Figure 3C). Also, surgery, radiation, and chemotherapy could prolong CSS of ASC cases (*p=*0.007, *p*=0.004, and *p*=0.014, respectively, Figure 3D-F).

Then, the prognostic factors associated with OS and CSS were further identified via univariate and multivariate Cox proportional hazard models in the ASC group. Table 2 summarized that distant summary stage and distant metastasis were significantly associated with poorer OS (*p*<0.05). Conversely, surgery, radiation, and chemotherapy were protective factors for OS (*p*<0.01 for all). The results of multivariate analyses demonstrated that distant summary stage (HR=2.11, *p*=0.014), surgery (HR=0.45, *p*=0.016), and chemotherapy (HR=0.30, *p*<0.001) remained independent risk factors for OS in gastric ASC patients. Interestingly, the lymph node metastasis was found to be a protective factor in univariate Cox analysis, however, there was no significant difference after multivariate analysis (*p*=0.054). For CSS analyses, Table 3 showed that ASC patients at distant summary stage had worse CSS in univariate analysis (*p*=0.008). And the CSS of ASC cases receiving surgery (*p*=0.012) and radiation (*p*=0.009), and chemotherapy (*p*=0.022) was longer. We found that chemotherapy (HR=0.45, *p*=0.018) could impact the CSS independently through multivariate analyses.

### Survival analyses in matched groups

**Figure 4:**
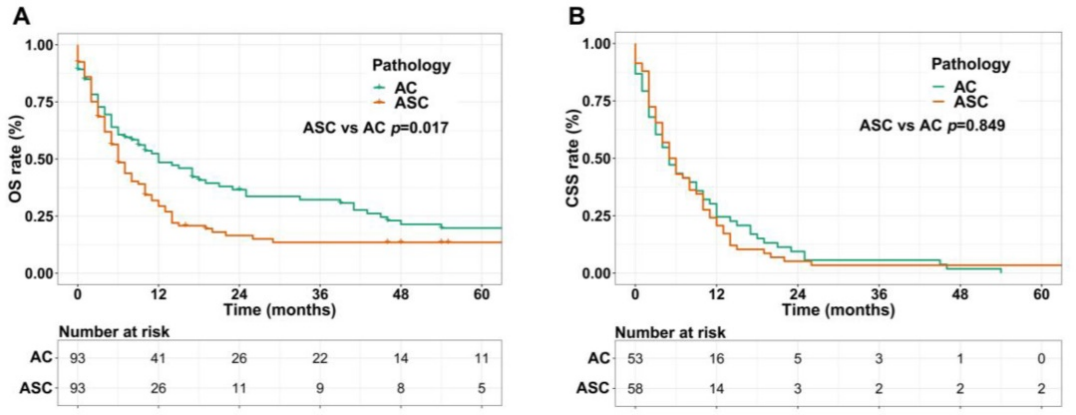
** (A)** OS for patients with gastric ASC and AC after PSM; **(B)** CSS for patients with gastric ASC and AC after PSM. ASC: adenosquamous carcinoma; AC: adenocarcinoma; PSM: propensity score matching.

**Figure 5:**
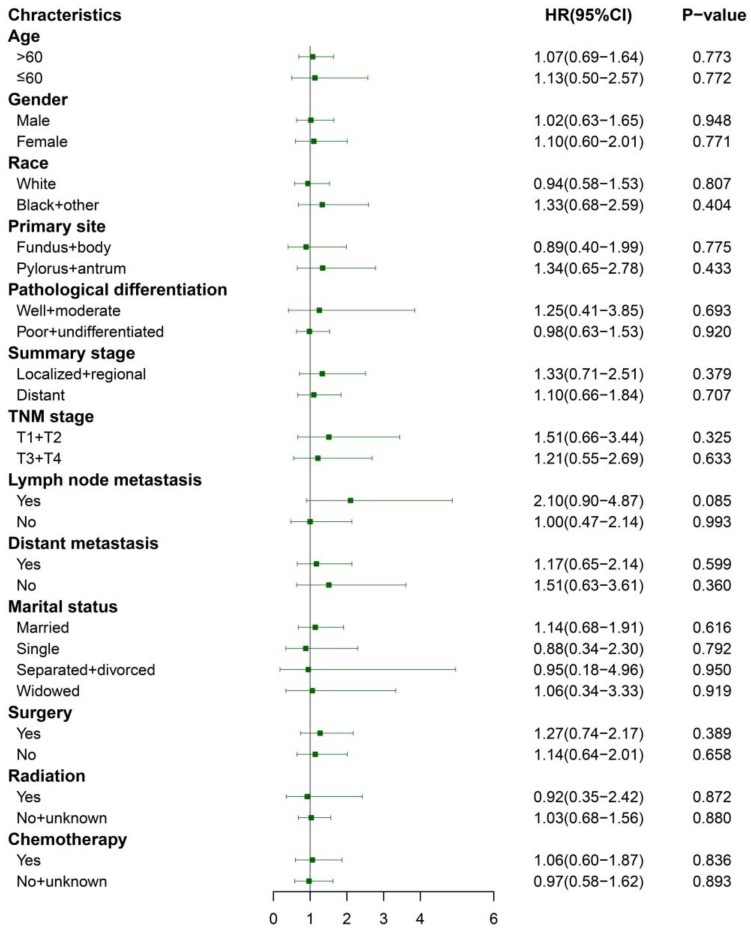
Subgroup analyses of CSS between the matched ASC and AC groups after PSM. ASC: adenosquamous carcinoma; AC: adenocarcinoma; PSM: propensity score matching.

We performed 1:1 matched analysis by PSM to match ASC patients with AC patients, balancing the differences of the baseline characteristics. A total of 186 gastric cancer patients consisting of 93 ASC cases and 93 AC cases were obtained after PSM and no significant differences were observed in basic clinical features (*p*>0.05 for all) (Table 4). Using the Kaplan Meier method, we still found that matched ASC patients had worse OS (*p*=0.017), but not CSS (*p*=0.849) than matched AC (Figure 4A and B). The subgroup analyses were carried out to elucidate whether the CSS between the ASC and AC groups existed differences. Regrettably, the results of univariate Cox analyses suggested no statistical difference between ASC patients and AC patients in all subgroups (*p*>0.05, Figure 5).

## Discussion

The incidence of gastric ASC is extremely low, only comprising <1% of all gastric malignancies. Most studies about gastric ASC were limited to case reports or small series, so our population-based study used data from SEER between 2000 and 2014 to better understand the clinicopathological features and prognosis of it. As expected, the results confirmed the rarity of gastric ASC (ASC: 0.2%, AC: 99.8%) again. It's worth noting that the incidences of ASC from 1983 to 2014 and AC from 1973-2014 decreased over time, which likely attributed to the prevention of H. pylori colonization, reduced salt intake, increased screening rate of gastroscopy, and so on.

In the present study, the average age of patients with gastric ASC was 68.7 years (range: 29 to 89 years), which was older than Feng et al.'s report with a mean age of 61.3 years 8. With regard to sex, our study indicated that the male to female ratio was 1.66, agreeing with Chen et al.'s result (3.3:1) and suggesting more males could be affected than females 6. In term of ethnicity, most gastric ASC patients belonged to the white race (60.2%), this was likely owing to the race distribution of western population. As for the primary site, we took Honda et al.'s study as reference 9, treating “antrum/pylorus” as synonymous with the lower third. Our results showed that the most common location of ASC was antrum/pylorus (35.5%), consistent with Feng et al. and Ajoodhea et al.'s reports 10. Although other researches revealed that lesions of most gastric ASC were located at the upper third of stomach 6, 11, 12, but they included few patients and lacked enough persuasion.

According to previous studies' results, gastric ASC, similar to the ASC occurring in other digestive system (such as esophagus, pancreas, and colon/rectum) 13-15, was extremely aggressive than AC. Our study also identified that patients with gastric ASC presented later summary stage, later T stage, and higher distant metastasis than those with AC. As we all known, gastric ASC was a mixed-pattern carcinoma of glandular and squamous components. Which kind of component mainly determined the malignant biological behavior of ASC, however, the results of previous studies remained inconsistent. Chen et al. 6 and Saito et al.'s 11 studies showed that both glandular and squamous components could lead to distant metastasis. Some other reports found that the biological behaviors of ASC were mainly determined by the AC component 16, 17. In all, the results based on single case report or small case series couldn't reach a convincing conclusion. Regrettably, our data from SEER database lacked the detail information about the component in metastatic lesions of ASC.

Due to the special etiology and biological features of ASC, gastric ASC patients carried a worse OS than AC patients before and after 1:1 matching ASC with AC using PSM, which was in accordance with previous studies 7, 8. In addition, this is the first study to evaluating the CSS between ASC patients and AC patients. However, for CSS analyses, whether we used PSM or not, the outcomes between these two pathology types showed no statistical differences. Therefore, we further performed the subgroup analyses and also found no statistical significance between ASC and AC patients in all subgroups. The possible explanation is that our study didn't had strong statistical power to detect the difference between ASC and AC cases due to too small sample size of ASC patients caused by the rarity of gastric ASC.

Then the risk factors for ASC patients' survival were analyzed, like Qin et al.' study 18, we included the SEER historic stage in the multivariate Cox analyses. The results revealed that distant summary stage was independently associated with poorer OS. As with gastric AC, tumor stage was the most significant prognostic factor. Yu et al.'s study observed that distal gastric cancer patients had a higher 5-year survival rate (51%) than proximal gastric cancer (28%) 19. In our present research, however, patients with ASC located at antrum/pylorus sites didn't have better OS. Furthermore, previous studies have shown that diagnosis of cancer led to more distress than other diseases 20 and psychological stresses influenced immune function, contributing to tumor progression and mortality 21. Married patients could obtain more social supports from their spouses or friends, in turn, displaying less distress and depression after the cancer diagnosis 22. Our Kaplan-Meier analyses showed that compared with married patients, single patients had shorter OS. Nonetheless, no significant difference was found when we conducted the Cox univariate analysis, which was likely caused by low statistical power or the different biological behaviors of gastric ASC from traditional AC. Because of ASC's rarity, no special standard therapeutic strategies were well established. Few studies indicated that the chemotherapy with S-1 (plus paclitaxel) could improve survival 1, 23. In our study, a remarkably longer OS was also observed in patients receiving surgery and chemotherapy after multivariate analyses. Chen et al.'s study 6 reported that adjuvant radiotherapy could improve survival time due to the squamous components of ASC. Our results also showed the similar trend, but without a statistically significant difference. For CSS of gastric ASC patients, our study indicated only chemotherapy predicted better survival independently.

A few hypotheses have been proposed regarding the origin of SCC component in ASC 24: (1) metaplastic transformation of AC; (2) cancerization of metaplastic squamous cells or ectopic squamous epithelium; (3) collision of AC and SCC; and (4) stem cell differentiation towards both glandular and squamous cells. However, the results remained controversial, and more studies are required to verify the histogenesis of gastric ASC.

Besides, similar with other retrospective studies using SEER as a data source, our study also had some limitations. Firstly, our results mainly illuminated the clinical features of ASC in California, not representing the patients in other areas. Secondly, we lacked clinicopathologic data (such as pathological differentiation, summary stage, T stage, lymph node metastases, and distant metastases) for some patients and didn't accurately describe the clinical parameters of gastric ASC. Thirdly, SEER database had no detailed chemotherapy regimens or information about targeted drugs, being not available to assess the impacts of specific treatment regimens. In the future studies, we will collect information of ASC patients from our own hospital, and further clarify the clinicopathologic characteristics of it.

## Conclusion

Gastric ASC was a unique and rare histological type in gastric cancer. Compared with AC patients, ASC cases presented poorer differentiation, later summary stage, later T stage, a higher distant metastasis rate, and worse OS before and after PSM, but not for CSS. More ASC patients received chemotherapy. Multivariate analyses showed that early summary stage, surgery, and chemotherapy may be favorable for OS for patients with gastric ASC. For CSS, chemotherapy could improve the outcomes of ASC patients.

## Figures and Tables

**Table 1:** Characteristics of patients with gastric ASC and AC

Characteristics	ASC	AC	*p* value
**Number**	93(0.2%)	41794(99.8%)	
**Age (years)**	68.72±12.42	70.85±15.19	0.078
**Gender**			
Female	35(37.6%)	17194(41.1%)	0.528
Male	58(62.4%)	24600(58.9%)	
**Ethnicity**			
White	56(60.2%)	26146(62.6%)	0.078
Black	25(26.9%)	7280(17.4%)	
Other	12(12.9%)	8262(19.8%)	
Unknown	0	106(0.3%)	
**Marital status**			
Single	14(15.1%)	5222(12.5%)	0.128
Married	58(62.4%)	22164(53.0%)	
Separated/divorced	6(6.5%)	3402(8.1%)	
Widowed	10(10.8%)	8837(21.1%)	
Other/unknown	5(5.4%)	2169(5.2%)	
**Primary site**			
Fundus	9(9.7%)	2403(5.7%)	0.155
Body	8(8.6%)	5244(12.5%)	
Antrum	26(28.0%)	12233(29.3%)	
Pylorus	7(7.5%)	1871(4.5%)	
Lesser curvature, NOS	14(15.1%)	4884(11.7%)	
Greater curvature, NOS	4(4.3%)	2197(5.3%)	
Overlapping lesion	13(14.0%)	4304(10.3%)	
Stomach, NOS	12(12.9%)	8658(20.7%)	
**Pathological differentiation**			
Well	1(1.1%)	1924(4.6%)	0.002
Moderate	11(11.8%)	11031(26.4%)	
Poor	69(74.2%)	21885(52.4%)	
Undifferentiated	2(2.2%)	637(1.5%)	
Unknown	10(10.8%)	6317(15.1%)	
**Summary stage**			
In situ	0	555(1.3%)	0.002
Localized	9(9.7%)	10009(23.9%)	
Regional	37(39.8%)	12785(30.6%)	
Distant	43(46.2%)	14026(33.6%)	
Unstaged	4(4.3%)	4419(10.6%)	
**T stage**			
T0/Tis/T1	7(7.5%)	7740(18.5%)	0.036
T2	19(20.4%)	8132(19.5%)	
T3	9(9.7%)	3428(8.2%)	
T4	14(15.1%)	3762(9.0%)	
Unknown	44(47.3%)	18732(44.8%)	
**Lymph node metastasis**			
Yes	32(34.4%)	11747(28.1%)	0.259
No	23(24.7%)	13233(31.7%)	
Unknown	38(40.9%)	16814(40.2%)	
**Distant metastasis**			
Yes	31(33.3%)	9970(23.9%)	0.026
No	28(30.1%)	17871(42.8%)	
Unknown	34(36.6%)	13953(33.4%)	
**Surgery**			
Yes	57(61.3%)	21113(50.5%)	0.080
No	36(38.7%)	20130(48.2%)	
Unknown	0	551(1.3%)	
**Radiation**			
Yes	19(20.4%)	6880(16.5%)	0.326
No/unknown	74(79.6%)	34914(83.5%)	
**Chemotherapy**			
Yes	41(44.1%)	14218(34.0%)	0.048
No/unknown	52(55.9%)	27576(66.0%)	

ASC: adenosquamous carcinoma; AC: adenocarcinoma. The significant *p* values are in bold.

**Table 2:** Univariate and Multivariate Cox proportional hazard analyses of clinical characteristics for OS in patients with gastric ASC

Factor	Category	Univariate		Multivariate	
		HR (95% CI)	*p* value	HR (95% CI)	*p* value
Age	>60 vs ≤60	1.24(0.70-2.17)	0.466		
Gender	Male vs Female	0.95(0.60-1.52)	0.841		
Ethnicity	White vs Black/other	0.74(0.46-1.17)	0.198		
Primary site	Antrum/pylorus vs Fundus/body	0.56(0.30-1.04)	0.067		
Pathological differentiation	Poor/undifferentiated vs Well/moderate	0.90(0.44-1.84)	0.779		
Summary stage	Distant vs Localized/regional	2.90(1.76-4.78)	<0.001	2.11(1.17-3.83)	0.014
T stage	T3/T4 vs T1/T2	0.89(0.47-1.68)	0.718		
Lymph node metastasis	Yes vs No	0.39(0.21-0.72)	0.003	0.49(0.24-1.01)	0.054
Distant metastasis	Yes vs No	2.69(1.46-4.94)	0.001	1.69(0.82-3.47)	0.156
Marital status	Single vs Married	1.98(1.00-3.91)	0.050		
	Separated/divorced vs Married	1.04(0.41-2.65)	0.922		
	Widowed vs Married	1.10(0.54-2.26)	0.794		
Surgery	Yes vs No	0.48(0.30-0.77)	0.002	0.45(0.24-0.86)	0.016
Radiation	Yes vs No/unknown	0.38(0.20-0.70)	0.002	0.78(0.40-1.52)	0.471
Chemotherapy	Yes vs No/unknown	0.42(0.27-0.67)	<0.001	0.30(0.17-0.52)	<0.001

ASC: adenosquamous carcinoma; AC: adenocarcinoma; OS: overall survival. The significant *p* values are in bold.

**Table 3:** Univariate and Multivariate Cox proportional hazard analyses of clinical characteristics for CSS in patients with gastric ASC

Factor	Category	Univariate		Multivariate	
		HR (95% CI)	*p* value	HR (95% CI)	*p* value
Age	>60 vs ≤60	1.05(0.54-2.04)	0.887		
Gender	Male vs Female	0.99(0.58-1.69)	0.979		
Ethnicity	White vs Black/other	0.89(0.52-1.51)	0.658		
Primary site	Antrum/pylorus vs Fundus/Body	1.00(0.49-2.05)	0.995		
Pathological differentiation	Poor/undifferentiated vs Well/moderate	0.55(0.24-1.27)	0.160		
Summary stage	Distant vs Localized/regional	2.16(1.23-3.81)	0.008	1.53(0.73-3.22)	0.262
T stage	T3/T4 vs T1/T2	1.00(0.48-2.05)	0.989		
Lymph node metastasis	Yes vs No	0.70(0.34-1.45)	0.335		
Distant metastasis	Yes vs No	1.58(0.80-3.10)	0.186		
Marital status	Single vs Married	2.03(0.95-4.34)	0.067		
	Separated/divorced vs Married	0.72(0.17-2.99)	0.647		
	Widowed vs Married	1.24(0.50-3.05)	0.641		
Surgery	Yes vs No	0.48(0.27-0.85)	0.012	0.50(0.23-1.12)	0.091
Radiation	Yes vs No/unknown	0.36(0.17-0.78)	0.009	0.66(0.28-1.58)	0.352
Chemotherapy	Yes vs No/unknown	0.54(0.31-0.91)	0.022	0.45(0.23-0.87)	0.018

ASC: adenosquamous carcinoma; AC: adenocarcinoma; CSS: cancer specific survival. The significant *p* values are in bold.

**Table 4:** Characteristics of patients with gastric ASC and AC after PSM

Characteristics	ASC	AC	*p* value
**Number**	93	93	
**Age (years)**	68.72±12.42	69.23±13.70	0.825
**Gender**			
Female	35(37.6%)	36(38.7%)	1.000
Male	58(62.4%)	57(61.3%)	
**Ethnicity**			
White	56(60.2%)	59(63.4%)	0.868
Black	25(26.9%)	22(23.7%)	
Other	12(12.9%)	12(12.9%)	
**Marital status**			
Single	14(15.1%)	14(15.1%)	0.779
Married	58(62.4%)	51(54.8%)	
Separated/divorced	6(6.5%)	10(10.8%)	
Widowed	10(10.8%)	13(14.0%)	
Other/unknown	5(5.4%)	5(5.4%)	
**Primary site**			
Fundus	9(9.7%)	9(9.7%)	0.580
Body	8(8.6%)	8(8.6%)	
Antrum	26(28.0%)	30(32.3%)	
Pylorus	7(7.5%)	2(2.2%0	
Lesser curvature, NOS	14(15.1%)	11(11.8%)	
Greater curvature, NOS	4(4.3%)	6(6.5%)	
Overlapping lesion	13(14.0%)	9(9.7%)	
Stomach, NOS	12(12.9%)	18(19.4%)	
**Pathological differentiation**			
Well	1(1.1%)	2(2.2%)	0.263
Moderate	11(11.8%)	14(15.1%)	
Poor	69(74.2%)	57(61.3%)	
Undifferentiated	2(2.2%)	1(1.1%)	
Unknown	10(10.8%)	19(20.4%)	
**Summary stage**			
In situ	0	1(1.1%)	0.746
Localized	9(9.7%)	11(11.8%)	
Regional	37(39.8%)	35(37.6%)	
Distant	43(46.2%)	39(41.9%)	
**Unstaged**	4(4.3%)	7(7.5%)	
**T stage**			
T1	7(7.5%)	12(12.9%)	0.663
T2	19(20.4%)	16(17.2%)	
T3	9(9.7%)	8(8.6%)	
T4	14(15.1%)	10(10.8%)	
Unknown	44(47.3%)	47(50.5%)	
**Lymph node metastasis**			
Yes	32(34.4%)	27(29.0%)	0.693
No	23(24.7%)	23(24.7%)	
Unknown	38(40.9%)	43(46.2%)	
**Distant metastasis**			
Yes	31(33.3%)	31(33.3%)	1.000
No	28(30.1%)	28(30.1%)	
Unknown	34(36.6%)	34(36.6%)	
**Surgery**			
Yes	57(61.3%)	54(58.1%)	0.765
No	36(38.7%)	39(41.9%)	
**Radiation**			
Yes	19(20.4%)	22(23.7%)	0.724
No/unknown	74(79.6%)	71(76.3%)	
**Chemotherapy**			
Yes	41(44.1%)	45(48.4%)	0.659
No/unknown	52(55.9%)	48(51.6%)	

ASC: adenosquamous carcinoma; AC: adenocarcinoma; PSM: propensity score matching.
